# A Survey of Ticks Infesting Dogs and Cats in Ireland

**DOI:** 10.3390/ani10081404

**Published:** 2020-08-12

**Authors:** Theo de Waal, Amanda Lawlor, Annetta Zintl, Bosco Cowley, Atiyah Bagha

**Affiliations:** 1 School of Veterinary Medicine, University College Dublin, D04 W6F6 Dublin, Ireland; amanda.lawlor@ucd.ie (A.L.); annetta.zintl@ucd.ie (A.Z.); atiyah.bagha@ucdconnect.ie (A.B.); 2 MSD Animal Health, D18 X5K7 Dublin, Ireland; bosco.cowley@merck.com

**Keywords:** ticks, companion animals, owner attitudes, Ireland

## Abstract

**Simple Summary:**

Ticks are important ectoparasites of companion animals not only causing localised skin damage, but are also vectors for a variety of pathogens. Veterinarians submitted ticks found on dogs and cats during routine examination for identification and completed a short questionnaire. A total of 120 ticks were submitted and we found that *Ixodes ricinus,* the sheep/castor bean tick, was the most abundant species on dogs while *Ixodes hexagonus*, the hedgehog tick, was the most abundant species on cats. Although most veterinary practitioners indicated that ticks were a concern to their clients, at the same time neither vets nor their clients were particularly concerned about tick-borne diseases in their animals. Interviews of dog walkers in the greater Dublin area indicate that pet owners are well aware of the presence of ticks in their surroundings. The loss of efficacy of some ectoparasiticides flagged by many pet owners requires further investigation.

**Abstract:**

Ticks are important ectoparasites of dogs and cats. Infestations can result in itching and localised dermatitis. In addition, ticks can act as vector of a range of viral, bacterial and protozoal pathogens. This paper reports the results of a nationwide survey of ticks infesting dogs and cats in Ireland. Seventy veterinary practices submitted a total of 120 ticks collected from 56 dogs and 16 cats. *Ixodes ricinus* was the most abundant species on dogs while *Ixodes hexagonus* was the most abundant species on cats. The remainder were identified as *Ixodes canisuga* and a single *Rhipicephalus sanguineus* specimen. The garden was most frequently associated with tick exposure in both dogs and cats. Sporting dog breeds (n = 17; 31%) were more likely to be infested with ticks than any other breed. Nearly all (n = 56; 95%) veterinarians indicated that ticks are a concern to their clients when they are found on their pets. Pet owners used a variety of products to control ectoparasites on their animals but a significant number (n = 18, 31%) indicated that they felt that the products are less effective highlighting the need for further investigations. Field sampling indicated that ticks are present at a low level in much of the greater Dublin area.

## 1. Introduction

Ticks on dogs and cats can severely damage the skin as a result of localised dermatitis and secondary bacterial infection. Heavy and repeated infestation may also lead to the development of anaemia and, rarely, immune suppression [1]. But the most significant consequence of tick infestation is the fact that they can also serve as vectors of a wide range of pathogens including *Babesia* spp., *Anaplasma* spp. and *Borrelia burgdorferi* [1,2] some of which infect humans as well as companion animals.

In Ireland dogs and cats are mostly infested by three tick species, *Ixodes ricinus* (sheep/castor bean tick)*, I. canisuga* (dog tick) and *I. hexagonus* (hedgehog tick) [3]. All three species are 3-host ticks meaning that each life cycle stage feeds on a different host, with blood digestion, moulting and oviposition taking place in the environment. While *I. ricinus* is known to transmit a variety of disease agents [1] the vector status of the other two species in Ireland is unknown.

Recent years have seen a significant increase in interest in ticks and tick-borne diseases both from a veterinary and medical point of view but also from the general public, highlighting the importance of the ‘One health’ approach to the problem [4]. The issue is compounded by the emergence of acaricide resistance which has been reported for tropical 1-host cattle ticks [5]. However, there is little information on resistance development in ticks collected from companion animals. The only exception is a study from Spain which reported high resistance rates in brown dog- or kennel ticks (*Rhipicephalus sanguineus*) to deltamethrin and variable sensitivity to propoxur [6]. At the time of writing there is no documented evidence of resistance in *Ixodes* spp. ticks in Europe.

As the most recent survey of tick infestation in dogs and cats in Ireland dates from 2000 [3] the main aim of this study was to investigate whether the prevalence of ticks infesting Irish pets has changed in the last two decades and, most importantly, whether new species have become established. Moreover, participating veterinary practitioners were asked whether they or their clients were concerned about ticks and tick-borne disease and the methods they used to control them. Finally, amenities in the greater Dublin area were visited to assess the likely risk from tick infestation for dogs and dog walkers. Although Dublin is located in a part of the country that, due to its drier climate and lack of suitable habitat, is considered less suitable for ticks [7], there is anecdotal evidence that pets (and joggers) may become infested when visiting certain sites. These sites were examined for the presence of ticks using standard blanket dragging methods and interviews of dog walkers.

## 2. Materials and Methods

### 2.1. Veterinary Practice-Based Tick Survey

Between 2016 and 2019, veterinary practices recruited through MSD Animal Health network in the Republic of Ireland and Northern Ireland were provided with a protocol on tick collection, sample tubes, a questionnaire (see Supplementary Materials) and a prepaid padded envelope for the return of samples and completed questionnaires.

No randomisation protocol was implemented when selecting participating veterinary practices or animals in the practice. Both dogs and cats were included in the study. The tick collection protocol required: (i) inspection of the head area with special attention given to the ears, particularly the inside and the area behind the ears and (ii) thorough examination of the neck and chest area, legs, armpits and between the toes. This was followed by (iii) brushing the fingers through the animal’s fur from head to tail and then from tail to head, applying enough pressure to detect any small lumps. Finally, (iv) a nit or flea comb was used to part the hair along the length of the body to check for ticks. The whole process took approximately 3 min. Any ticks that were found were removed using a tick hook or tweezers, ensuring the mouthparts remained attached to the tick and intact. For operational reasons and to encourage maximum participation it was requested that only up to five ticks per animal be collected. Collected ticks were placed into individual tubes labelled with the animal’s name and collection date and stored at −20 °C until they were posted to the University College Dublin, School of Veterinary Medicine. On arrival in the laboratory each sample was given a unique identification number. Ticks were identified to species level, life cycle stage and sex using various identification keys [8,9], (http://www.bristoluniversitytickid.uk). All tick identifications were cross-checked by a second investigator. In cases where more than five ticks per animal were submitted only the first five ticks were included in the study to avoid bias.

The questionnaire data including age, sex, breed, the type of habitat the animal had visited in the previous two weeks and recent travel history were entered into Google sheets (Google LLC, Mountain View, CA, USA). General concerns of veterinarians and owners with regard to ticks, the incidence of tick-borne disease and the tick control measures they used were also noted. All data analysis was performed using EpiInfo 7 (https://www.cdc.gov/epiinfo/index.html).

### 2.2. Field Survey for the Presence of Ticks in the Greater Dublin Area

Between May and June 2019, nine amenities in the greater Dublin area, including Howth Cliff Path Loop, Marlay Park, Mount Pelier Hill (locally known as the Hellfire Club), Phoenix Park, Bushy Park, Blackrock Park, St. Anne’s Park, Killiney Hill, and Bull Island, were investigated for the presence of ticks using standard blanket dragging methods and interviews of dog walkers. In each amenity, five locations of potentially suitable tick habitat (i.e., woodland, rough scrub and hedges) were examined (with 35 × 5 m sweeps in each) and 10 dog walkers were interviewed.

## 3. Results and Discussion

### 3.1. Veterinary Practice-Based Tick Survey

**Figure 1:**
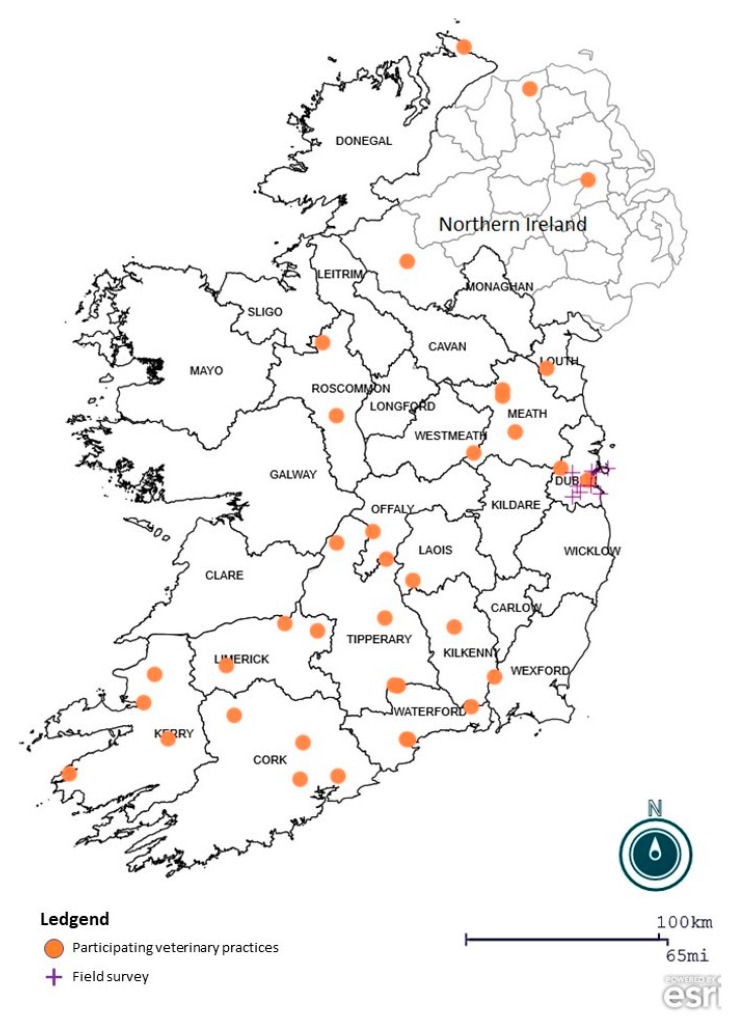
Distribution of participating veterinary practices from which submission were received as well as the field collection sites.

A total of 70 veterinary practices from 17 counties across Ireland participated in the survey (Figure 1), 3 practices did not indicate their county. Overall, 120 ticks from 56 dogs and 16 cats, were submitted together with completed questionnaires. All of the submitted ticks were adults of which 116 were female and 4 male. A median number of 1 tick was estimated by veterinarians to be present on both dogs and cats but overall estimation of infestation levels ranged from 1 to 100 on dogs and 1 to 40 on cats. Compared with similar studies elsewhere, participation in the survey was low. For example, a study in the UK, ‘The Big Tick Project’ attracted over 1000 participating practices who submitted over 6000 ticks, however, these researchers used radio, television, print and social media to raise awareness for their campaign [10].

The tick species identified in the survey are summarised in Table 1. As previously reported with regard to pets in the UK and Ireland [3,10], almost all ticks were identified as *Ixodes* species, namely *I. ricinus*, *I. canisuga* and *I. hexagonus*. Only 2 animals were infested with more than one tick species. *I. ricinus* was the most common tick found on dogs (n = 33; 59%) and *I. hexagonus* the most common tick on cats (n = 8; 53%). It is thought that this discrepancy is due to the different hunting behaviours of dogs and cats. *I. ricinus* is an exophilic tick, i.e., it lives freely in the environment and actively seeks hosts, which may increase the potential exposure of dogs. In contrast, both, *I. hexagonus* and *I. canisuga* are generally considered nidicolous, i.e., they remain in or adjacent to their hosts’ burrows and nests which may be more frequently visited by cats. In adult *I. hexagonus* ticks this lifestyle may to be adhered to less stringently, as they are occasionally detected at some distance from their host’s nests. Surprisingly, we also identified a single *R. sanguineus* sensu lato tick on one dog from county Dublin. *R. sanguineus* is more commonly found in warmer climates and there are only two unconfirmed records of *R. sanguineus* from Belfast in 1968 and 1981, respectively (https://species.nbnatlas.org/ species/NBNSYS0000039964). Therefore, our finding is difficult to explain, especially since this dog did not have travel history outside its home location and warrants further investigation.

With regard to tick-borne diseases, *I. ricinus* potentially poses the greatest risk to animal (and human) health as it serves as a vector for *B. burgdorferi*, *A. phagocytophilum*, Louping ill virus and a number of *Babesia* spp.—*B. divergens, B. venatorum* and *B. microti*; although none of these *Babesia* spp. infects cats or dogs. Less is known about the vectorial capacity of both *I. hexagonus* and *I. canisuga*, and to date there is only experimental evidence showing that both these tick species can serve as vectors of *B. burgdorferi* [9,11]. Nothing is known about the importance of these tick-borne diseases in dogs and cats in Ireland as no extensive surveys have yet been conducted in this region. There is one unpublished report of *A. phagocytophilum* in cats from Ireland, cited by Juvet et al. [12]. Juvet et al. [12] did not detect *Ehrlichia* or *Anaplasma* species DNA in blood samples from 121 cats collected in the greater Dublin area.

The majority of dogs and cats (60%) were only exposed to a garden habitat in the two weeks prior to tick collection (Table 2). None of the animals had travelled abroad in the past two weeks. Only 4 dogs had travelled within Ireland and all of them were infested with *I. ricinus*.

As would be expected, sporting dog breeds (n = 17; 31%) were more likely to be infested with ticks and the most prevalent species found on these dogs was *I. ricinus* (n = 11; 65%) (Table 3). There was little difference in tick infestation prevalence between working dogs (46%; n = 16) and city pets (54%; n = 19) highlighting the fact that, in areas where ticks occur, pets may be exposed to ticks regularly even if they do not stray far from their home environment.

Nearly all (n = 56; 95%) veterinary practitioners that took part in the survey indicated that ticks were a concern to their clients when they find them on their pets. At the same time neither vets (n = 37; 64%) nor their clients (n = 31; 54%) were particularly concerned about tick-borne disease which is probably due to the fact that most of the tick-borne pathogens that are known to occur in Ireland are not infectious to dogs or cats. Only four (8%) practices reported diagnosis of a tick-borne disease in the last year and only two of these specified the tick-borne disease, both as ehrlichiosis. However, it is not clear which *Ehrlichia* spp. may have been involved as this pathogen has never been reported from dogs in Ireland. Although its tick vector, *R. sanguineus*, was reported in Northern Ireland (https://species.nbnatlas.org/species/NBNSYS0000039964), it is highly unlikely that this tick species is established on the island. On the other hand, *A. phagocytophilum* which is transmitted by *I. ricinus*, includes strains that are specific to ruminants and strains that infect horses, dogs and humans. While the strains of *A. phagocytophilum* infecting ruminants appears to be widespread on the island [13], it is not known whether strains infecting dogs occur here.

**Figure 2:**
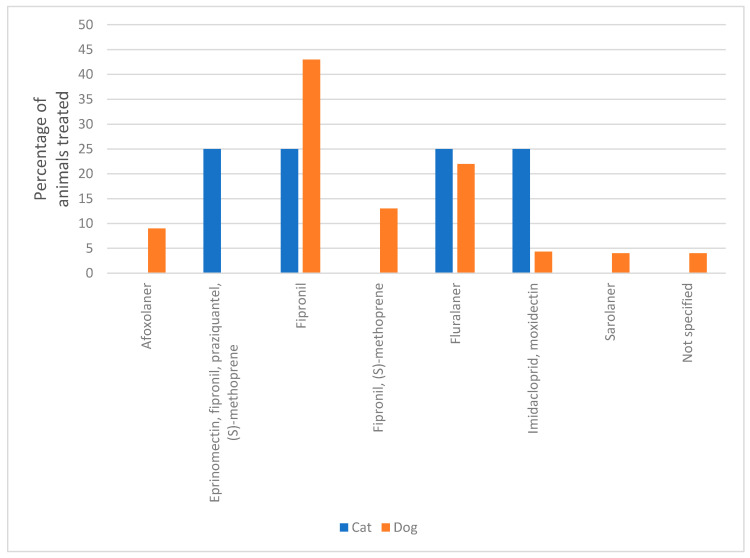
Active compounds of the ectoparasiticides used to treat dogs and cats in the survey.

Only 22 of the dogs and four of the cats were recently treated for ticks using a range of active compounds listed in Figure 2. While the majority of pet owners (n = 34; 57%) did not know if there was any change in effectiveness of the compounds, 31% (n = 18) indicated that they felt that the product was less effective (Table 4) than it was previously. This is an interesting observation as there is currently no documented evidence of acaricide resistance in *Ixodes* spp. ticks in Europe and warrants further investigation.

### 3.2. Field Survey for the Presence of Ticks in the Greater Dublin Area

Blanket dragging revealed the presence of *I. ricinus* ticks in two sites, Mount Pelier Hill and Marlay Park, however, only a single nymph was collected in the latter park and this specimen was in poor condition and did not seem to be part of a viable population. In contrast a total number of 33 nymphs were collected in several locations on Mount Pelier Hill which, of the Dublin mountains, is the one closest to the city and a popular public amenity. The dog walkers confirmed the presence of ticks in Mount Pelier Hill (with nine out of 10 stating that they knew they were present there), and the absence of ticks in all the other amenities the exception of Howth Cliff Path where one dog walker claimed to have seen evidence of ticks, a finding that is worth exploring further.

The field survey was carried out during the period of peak tick activity in Ireland [14]. Blanket dragging is the standard method used to determine the presence of ticks in a site, however, its efficacy is dependent on tick activity and affected by temperature and humidity. In order to definitely rule out the presence of ticks in an area it is essential to revisit it and at times when the weather is conducive. It is also important to remember that only ticks that exhibit questing behavior can be caught using blanket dragging.

## 4. Conclusions

This study confirms findings of previous studies that the main tick species infesting dogs and cats in Ireland are *I. ricinus*, *I. hexagonus* and *I. canisuga*. While the risk from tick-borne disease is likely to remain low for dogs or cats, repeated infestations should prompt preventive measures if only to reduce the likelihood of direct injury. The loss of efficacy of some ectoparasiticides over time, flagged by pet owners requires further investigation. When determining the presence or absence of ticks in a given site, local knowledge provides a useful addition to standard field survey techniques.

## Figures and Tables

**Table 1:** Tick species detected on dogs and cats.

Tick Species	Dogs		Cats	
	n	Percent	n	Percent
*Ixodes canisuga*	13	23.21%	1	6.67%
*Ixodes hexagonus*	9	16.07%	8	53.33%
*Ixodes ricinus*	33	58.93%	6	40.00%
*Rhipicephalus sanguineus*	1	1.79%	-	-
Total	56	100.00%	15	100.00%

**Table 2:** Habitat exposure of dogs and cats in the two weeks prior to tick collection.

Exposed Habitat	Dogs		Cats	
	n	Percent	n	Percent
Farm Pasture	9	18.00%	4	26.67%
Garden	30	60.00%	9	60.00%
Urban Park	3	6.00%	0	-
Woodlands	8	16.00%	2	13.33%
Total	50	100.00%	15	100.00%

**Table 3:** Ticks detected on various dog breeds.

Tick Species	Kennel Club Breed Classification									
	Crossbred	Herding	Hound	Miscellaneous Class	Non-Sporting	Sporting	Terrier	Toy	Working	**Total**
*Ixodes canisuga*	0	2	5	3	0	2	1	0	0	13
*Ixodes hexagonus*	1	0	2	0	0	4	1	1	0	9
*Ixodes ricinus*	0	3	1	2	2	11	6	5	2	32
*Rhipicephalus sanguineus*	0	0	0	0	0	0	0	1	0	1
Total	1	5	8	5	2	17	8	7	2	55
	1.82%	9.09%	14.55%	9.09%	3.64%	30.91%	14.55%	12.73%	3.64%	100.00%

**Table 4:** Owners perception on the past and present effectiveness of ectoparasiticides used on dogs and cats.

Change in Effectiveness	n	Percent
Don’t know	34	57.63%
No, no change	7	11.86%
Yes, less effective	18	30.51%
Total	59	100.00%

## Supplementary Materials

The following are available online at https://www.mdpi.com/2076-2615/10/8/1404/s1, File S1: The Big Tick Hunt: cat & dog questionnaire.
