# Supplementary material for: A Survey of Ticks Infesting Dogs and Cats in Ireland
Source: Animals (Basel). 2020 Aug 12;10(8):1404. doi: 10.3390/ani10081404 (PMC7460173; doi:10.3390/ani10081404)
Supplement: Supplementary file 1 [file animals-10-01404-s001.pdf]

animals

MDPI

Supplementary Materials: A Survey of Ticks Infesting Dogs and Cats in Ireland

Theo de Waal [1,*], Amanda Lawlor [1], Annetta Zintl [1], Bosco Cowley [2] and Atiyah Bagha [1]

File S1. The Big Tick Hunt: cat & dog questionnaire.

Thank you for your time taken to participate in this survey; if you collected any ticks, please place them in a tube, label the tube with your Name and Address and the pet's name and store in the freezer. Please use a separate tube and questionnaire for each pet from which ticks were collected. At the end of each week, please send the questionnaires and collected ticks (from the freezer) to the address below using one of the pre-paid envelopes provided.

1. Name and address of practice

   ____

   ____

   ____

2. Which would best describe your practice?

   ☐ Small animal ☐ Large animal ☐ Mixed

3. Date of collection:____________(dd/mm) Pet's name ______________________________

4. Breed:______________________Age:______________Sex: ______________

5. Ticks Present? Yes ☐ No ☐ Estimated number of Ticks present: ______________

6. What habitats has the animal been exposed to in the previous two weeks?:

   Garden ☐ Urban Park ☐ Farm Pasture ☐

   Woodland ☐ Hillside-Moorland ☐ Beach ☐

7. Working farm dog ☐ City pet ☐

8. Has the dog been in kennels in the past two weeks? Yes ☐ No ☐

9. Has the dog been abroad in the past two weeks? Yes ☐ No ☐

10. Has the dog travelled elsewhere in Ireland/Northern Ireland in the past two weeks?

    Yes ☐ No ☐

If yes, where?

____

animals

MDPI

11. When was the dog last treated for ticks?______________________________(dd/mm/yy)

12. What acaricide was used in treatment?

____

____

13. Do you think there has been a change in the effectiveness of acaricides in recent years?

- ☐ Yes, they seem to be less effective now.
- ☐ No, there is no change in their effectiveness.
- ☐ Don't know.

14. Did you diagnose any tick borne diseases in your practice over the last year?
If yes please list them below and include the host species

____

____

15. Are ticks a concern for your clients?

- ☐ Yes, when they find them on their pets
- ☐ Yes, as potential vectors of human disease
- ☐ No, they do not seem to be concerned about ticks
- ☐ Don't know

16. How would you rate your concerns regarding risks posed by ticks?

- ☐ Very concerned
- ☐ Moderately concerned
- ☐ Mildly concerned
- ☐ Not concerned at all
- ☐ Don't know

17. How would you rate your client's awareness and concerns regarding risks posed by ticks?

- ☐ Very concerned
- ☐ Moderately concerned
- ☐ Mildly concerned
- ☐ Not concerned at all
- ☐ Don't know

Return address for samples and questionnaires:

**FOR ADDITIONAL SUPPLIES OR ANY OTHER QUERIES,
PLEASE SEND AN EMAIL TO:**
